# Study of risk management for orthodontists’ practice in Taiwan

**DOI:** 10.1016/j.jds.2025.02.010

**Published:** 2025-02-25

**Authors:** Sheng-Huai Wang, Johnson Hsin-Chung Cheng, Kuan Chung Huang, Daniel De-Shing Chen

**Affiliations:** a School of Dentistry, College of Oral Medicine, Taipei Medical University, Taipei, Taiwan; b Orthodontic Division, Department of Dentistry, Taipei Medical University Hospital, Taipei, Taiwan

**Keywords:** Clinical practice, Demographics, Orthodontics, Risk management, Taiwan

## Abstract

**Background/Purpose:**

Risk management in the health-care industry is a systematic approach for mitigating potential risks and enhancing patient safety. This study examined orthodontists’ perceptions of and attitudes toward risk management in Taiwan. The results provide clinical references informing policies for health-care institutions in Taiwan.

**Materials and methods:**

During the 2021 Taiwan Association of Orthodontics (TAO) annual meeting, a structured questionnaire was distributed to 143 randomly selected orthodontists. This questionnaire comprised 23 questions on demographics (5 questions), knowledge of risk management (5 questions), execution of and attitude toward risk management (8 questions), and adverse event experiences (7 questions). After the frequency distribution of each question was examined, demographics and participants’ preferences were analyzed through chi-square and multiple regression tests.

**Results:**

Goodness-of-fit testing revealed no significant differences in sex or age between the participants and TAO members. Most of the orthodontists were employees (68.31%). Additionally, most of the orthodontists (90.14%) believed that a favorable doctor–patient relationship and high-quality orthodontic treatment are essential for mitigating the risks associated with orthodontic care. Moreover, 30.28% of the orthodontists reported AEs, and 38.73% reported foreign body ingestion by patients. No significant correlation was observed between demographics and risk management.

**Conclusion:**

The majority of orthodontists in Taiwan have a positive understanding of and attitude toward risk management. Factors such as sex, age, and clinical experience have a minimal influence on the implementation of risk management practices by Taiwanese orthodontists. These findings provide valuable clinical references for governmental organizations aiming to formulate policies related to orthodontic practice and risk management.

## Introduction

Risk management is regarded as a key concern in medical practice worldwide. In 1965, the Centers for Medicare & Medicaid Services in the United States set certain standards for patient safety, including infection control and medication management.^1^ In 2005, the Patient Safety and Quality Improvement Act was implemented, encouraging clinicians to report medical errors and adverse events under legal protection.^2^ In the United Kingdom, the National Health Service established the Patient Safety Strategy, which emphasizes a “zero tolerance” approach to preventable errors, with a focus on system improvement and staff training.^3^

Many countries, including the United States,^4^ the United Kingdom,^5^ and Germany,^6^ have implemented preventive measures for dental risk management, including infection control, radiation safety, and surgical safety. The guidelines outlined by national dental associations focus on minimizing the risks associated with dental procedures. They also recommend the implementation of stringent protocols for infection prevention, safe use of X-ray equipment, and adherence to surgical safety standards to protect patients and health-care professionals.

Multiple professional dental organizations have recommended risk management in dentistry. Various specialist associations, including the American Association of Orthodontists, British Orthodontic Society, German Society of Orthodontics and Dentofacial Orthopedics, and Japanese Orthodontic Society, have established relevant guidelines.^7^, ^8^, ^9^ In Taiwan, specific regulations for risk management remain limited, and few regulations have been established for orthodontics. Therefore, this study examined orthodontists’ perceptions of and attitudes toward risk management in Taiwan. The results provide a valuable reference for establishing clinical guidelines for orthodontic practice in Taiwan.

## Materials and methods

This study was approved by the Institutional Review Board of Taipei Medical University Hospital (approval no. N202309067).

In this study, an anonymous academic questionnaire survey was conducted. This questionnaire was collaboratively developed by three researchers, comprising the coauthor and two other authors. After reviewing more than 50 articles, the authors developed a questionnaire that closely aligned with the research topic and current scenario in Taiwan. The questionnaire consisted of four parts: demographics (18 questions), perceptions of risk management (5 questions), attitudes toward risk management (11 questions), and experiences with adverse events (10 questions). A pretest (kappa coefficient) was conducted to determine the reliability of the questionnaire. The results indicated a kappa coefficient of 0.71, indicating substantial agreement.

After the pretest, participants were randomly selected from the 2021 Taiwan Association of Orthodontics (TAO) annual meeting. Before data collection, each participant was provided with a detailed description of the study. Subsequently, informed consent was obtained from each participant. Data were collected only from those who agreed to participate and signed a consent form.

Goodness-of-fit tests were conducted to identify statistically significant differences between the participants and TAO members. After the frequency distribution (%) of the questionnaire responses was calculated, the correlation between demographics and risk management was determined using a statistical analysis. Chi-square tests, t tests, and multiple linear regression tests were conducted to analyze orthodontists’ perceptions, attitudes, and adverse events. All statistical analyses were conducted using IBM SPSS Statistics version 19 (IBM, Armonk, NY, USA). A P value of 0.05 or less was considered statistically significant.

## Results

### Demographics

A total of 143 orthodontists completed the questionnaire at the 2021 TAO annual meeting. Table 1 presents the demographics of the participants. Most of the participants (68.3%) were employees, of whom 40.1% had fewer than 50 active treatment cases. In addition, 45.7% of the participants had fewer than 10 years of clinical experience. A goodness-of-fit test was conducted to identify significant differences between the participants and TAO members. No significant differences were observed in sex or clinical practice between the participants and TAO members. However, a significant difference was observed in age (P < 0.05).

**Table 1:** Frequency distribution of orthodontists’ demographics.

Demographics	Frequency	Distribution
Sex		
Female	77.0	54.23%
Male	65.0	45.77%
Age (years)		
31–40	66.0	46.48%
41–50	39.0	27.46%
51–60	28.0	19.72%
61–70	6.0	4.23%
>71	3.0	2.11%
Clinical experience (years)		
<5	29.0	20.42%
6–10	36.0	25.35%
11–15	19.0	13.38%
16–20	20.0	14.08%
>21	38.0	26.76%
Employment status		
Employee	97.0	68.31%
Employer	45.0	31.69%
Active treatment cases (n)		
<50	57.0	40.14%
51–100	49.0	34.51%
101–200	19.0	13.38%
>200	17.0	11.97%

## Perceptions

Table 2 presents the frequency distribution of the participants’ perceptions of risk management. For 73.94% of the participants, dental conferences were the primary source of information on risk management. However, 46.48% of the participants reported never attending such conferences. In terms of compliance with regulations regarding orthodontic chart retention in Taiwan, 72.54% of the participants provided correct responses. In addition, 86.62% agreed that patient dissatisfaction with orthodontic outcomes was a prevalent cause of adverse events in Taiwan.

**Table 2:** Frequency distribution of orthodontists’ perceptions of risk management.

Risk perceptions	Frequency	Distribution
Main sources of information		
Dental conferences	105.0	73.94%
Dental publications	18.0	12.68%
Internet	13.0	9.15%
Other	6.0	4.23%
Frequency of participation in continual education		
0 time	66.0	46.48%
1 time	57.0	40.14%
2 times	9.0	6.34%
3 times or more	10.0	7.04%
Orthodontic chart retention period		
5 years	13.0	9.15%
7 years	103.0	72.54%
9 years	4.0	2.82%
11 years or more	22.0	15.49%
Risk management of adverse events		
All of the above	128.0	90.14%
Favorable doctor–patient relationship	12.0	8.45%
High-quality and complete orthodontic records	2.0	1.41%
Adverse events of orthodontics in Taiwan		
Patient dissatisfaction with orthodontic outcomes	123.0	86.62%
Prolonged orthodontic time	11.0	7.75%
Inflammation due to invasive treatment	3.0	2.11%
Malpractice of tooth extraction	3.0	2.11%

### Attitudes

Table 3 provides the frequency distribution of the participants’ attitudes toward risk management. Most of the participants (69.72%) reported informing their patients of all risks, and 95.07% informed their patients of the risks of relapse and retention. In addition, 70.42% of the participants obtained informed consent from every patient, and 80.99% collected data both before and after treatment. In cases of unsuitable aligner therapy, 92.96% of the participants preferred to switch to fixed appliances.

**Table 3:** Frequency distribution of orthodontists’ attitudes toward risk management.

Attitudes toward risk management	Frequency	Distribution
Notification of potential risks		
Every patient	99.0	69.72%
Depending on severity	41.0	28.87%
Never	2.0	1.41%
Notification of risks of relapse and retention		
Every patient	135.0	95.07%
Depending on severity	7.0	4.93%
Frequency of obtaining informed consent		
0%–25%	18.0	12.68%
26%–50%	6.0	4.23%
51%–75%	4.0	2.82%
76%–99%	14.0	9.86%
100%	100.0	70.42%
Orthodontic records before and after treatment		
Before and after treatment	115.0	80.99%
Before or after treatment	18.0	12.68%
Before treatment only	9.0	6.34%
Response to difficult treatments		
Partial cooperation	76.0	53.52%
Treatment refusal	31.0	21.83%
Referral to another orthodontist	19.0	13.38%
Strict adherence to the doctor's plan	13.0	9.15%
Complete cooperation	3.0	2.11%
Response to unsuitable aligner therapy		
Combination of aligner and fixed appliances	89.0	62.68%
Use of fixed appliances	43.0	30.28%
Referral to another orthodontist	10.0	7.04%
Oral hygiene care		
No brushing required	66.0	46.48%
Brushing required	46.0	32.39%
Brushing noted and treatment continued	27.0	19.01%
Hygiene ignored and treatment continued	3.0	2.11%
Attitude toward orthodontic treatment		
Depending on the patient's condition	85.0	59.86%
Seeking perfection	30.0	21.13%
Achieving acceptable results	18.0	12.68%
Based on the patient's acceptance	9.0	6.34%

### Clinical experience

Table 4 presents a summary of the frequency distribution of adverse events. More than 30.28% of the participants reported adverse events, and 17% reported previous monetary settlements. More than one-third of the participants (38.73%) encountered cases of foreign body ingestion. In terms of misdiagnoses or incorrect treatment plans by other orthodontists, 54.93% of the participants informed their patients that their treatment was incomplete, and 88.73% referred their patients back to their original orthodontist. In addition, 26.06% of the participants were not part of a dental insurance plan.

**Table 4:** Frequency distribution of orthodontists’ experiences of adverse events.

Experience of adverse events	Frequency	Distribution
Frequency of adverse events		
0 time	99.0	69.72%
1 time	18.0	12.68%
2 times	11.0	7.75%
3 times	7.0	4.93%
Uncertain	7.0	4.93%
Frequency of monetary settlements		
0 time	117.0	82.39%
1 time	18.0	12.68%
2 times	6.0	4.23%
3 times	1.0	0.70%
Referred difficult cases		
0 case	64.0	45.07%
1–3 cases	37.0	26.06%
4–6 cases	14.0	9.86%
7–9 cases	4.0	2.82%
>10 cases	23.0	16.20%
Frequency of foreign body ingestion		
0 time	87.0	61.27%
1 time	18.0	12.68%
2 times	8.0	5.63%
3 times	6.0	4.23%
>4 times	23.0	16.20%
Response to misdiagnosis and incorrect treatment plans by others		
Only inform the patient that the treatment is in progress and not yet completed	78.0	54.93%
Do not explain too much	52.0	36.62%
Honestly inform the patient that the diagnosis and treatment plan are incorrect	12.0	8.45%
Follow-up actions		
Refer the patient back to the original orthodontist	126.0	88.73%
Take over the case and treat again	9.0	6.34%
Refer the patient to another orthodontist	7.0	4.93%
Dental insurance		
Yes	100.0	70.42%
No	37.0	26.06%
Uncertain	5.0	3.52%

### Correlation between demographics and risk management

The chi-square test (Table 5) revealed no significant correlation between most demographic factors and risk management. Similarly, no significant associations were found between remaining demographic factors and sources of information on risk management. Attendance at dental conferences, as a source of information, also showed no significant correlation with any demographics. Informing patients of potential risks was not significantly correlated with most demographic factors, except for a notable association between obtaining informed consent and sex. However, no significant correlations were observed for other demographics, such as age, clinical experience, clinical director status, or the number of active treatment cases (P > 0.05). Multiple regression analysis (Table 6) corroborated these findings, showing no significant relationships between most demographics and risk management. However, informing patients of potential risks was significantly associated with clinical experience, employment status, and the number of active treatment cases. Conversely, aligner use was not significantly correlated with any demographic factors.

**Table 5:** Correlation between demographics and risk management through chi-square testing.

	Sex	Age	Clinicalexperience	Employment status	Activetreatment cases
Main sources of information	5.319 (0.150)	13.379 (0.573)	17.175 (0.143)	3.327 (0.344)	10.701 (0.297)
Risk management of adverse events	3.457 (0.063)	10.871 (0.054)	1.173 (0.882)	1.224 (0.269)	3.822 (0.281)
Adverse events of orthodontics in Taiwan	2.110 (0.716)	27.219 (0.129)	22.184 (0.137)	4.856 (0.302)	13.659 (0.323)
Notification of potential risks	0.000 (1.000)	9.548 (0.089)	4.157 (0.385)	2.115 (0.146)	0.632 (0.889)
Notification of relapse and retention risks	0.000 (1.000)	4.256 (0.513)	7.922 (0.095)	0.411 (0.521)	0.141 (0.987)
Frequency of obtaining informed consent	13.652 (0.009∗)	30.104 (0.068)	8.300 (0.939)	5.860 (0.210)	13.973 (0.302)
Response to unsuitable aligner therapy	6.514 (0.089)	27.634 (0.024∗)	15.036 (0.239)	2.972 (0.396)	7.727 (0.562)
Oral hygiene care	2.620 (0.454)	9.713 (0.837)	14.898 (0.247)	9.216 (0.026∗)	14.169 (0.116)
Frequency of adverse events	4.146 (0.529)	36.044 (0.071)	36.902 (0.012∗)	3.418 (0.636)	9.326 (0.860)
Frequency of monetary settlements	7.610 (0.055)	27.160 (0.028∗)	30.548 (0.002∗∗)	6.501 (0.090)	19.587 (0.021∗)
Referred difficult cases	4.170 (0.384)	14.598 (0.799)	12.556 (0.705)	6.978 (0.137)	11.538 (0.483)
Frequency of foreign body ingestion	2.107 (0.716)	23.963 (0.244)	41.132 (0.001∗∗)	3.395 (0.494)	21.908 (0.039∗)

χ2(P): χ2 = Chi-square value; *P* = *P* value.

**Table 6:** Multiple regression of the correlation between risk management and orthodontists’ demographics.

	Sex	Age	Experience	Employmentstatus	Activetreatment cases
Main sources of information	0.221 (0.044∗)	0.069 (0.375)	−0.088 (0.121)	0.04 (0.754)	−0.011 (0.84)
Risk management of adverse events	0.956 (0.090)	1.017 (0.720)	0.094 (0.050)	0.061 (0.027∗)	0.062 (0.010∗)
Adverse events of orthodonticsin Taiwan	2.454 (0.015∗)	0.429 (0.669)	0.038 (0.201)	0.119 (0.072)	0.051 (0.078)
Notification of potential risks	−1.018 (0.310)	−1.73 (0.086)	−0.159 (0.045∗)	0.045 (0.020∗)	−0.078 (0.002∗)
Notification of relapse andretention risks	0.841 (0.148)	0.434 (0.408)	−0.205 (0.148)	0.019 (0.148)	0.016 (0.148)
Frequency of obtaining informedconsent	2.424 (0.192)	1.184 (0.191)	0.107 (0.192)	0.031 (0.192)	0.037 (0.192)
Response to unsuitable alignertherapy	−1.621 (0.337)	0.919 (0.337)	0.084 (0.337)	0.129 (0.337)	0.119 (0.337)
Oral hygiene care	0.705 (0.409)	−0.309 (0.409)	0.031 (0.409)	0.137 (0.409)	−0.234 (0.409)
Frequency of adverse events	1.111 (0.564)	0.164 (0.564)	0.015 (0.564)	0.298 (0.564)	0.049 (0.564)
Frequency of monetary settlements	−1.879 (0.620)	0.101 (0.620)	−0.012 (0.620)	0.12 (0.620)	−0.007 (0.620)
Referred difficult cases	−2.125 (0.294)	−0.338 (0.294)	−0.176 (0.294)	0.208 (0.294)	−0.07 (0.294)
Frequency of foreign body ingestion	−1.033 (0.464)	−0.245 (0.464)	−0.023 (0.464)	0.147 (0.464)	−0.036 (0.464)

β(P): β = regression coefficient; *P* = *P* value.

## Discussion

In this study, no significant differences were observed in the distribution of sex or clinical experience between the surveyed orthodontists and TAO members. In terms of age, 66% of the participants were aged under 40 years, greater than the corresponding proportion of TAO members (41.4%). By contrast, only 9% of the participants were aged above 60 years, lower than the corresponding proportion of TAO members (16.2%). Chi-square testing revealed a significant difference in age distribution (P < 0.001) between the participants and TAO members, presumably because older orthodontists, despite receiving invitations, did not participate in the survey. This nonresponse may have introduced bias in data collection, which is regarded as a limitation of this study. Moreover, 25.3% of the participants reported managing more than 100 active treatment cases each year. Data from the American Association of Orthodontists^22^ indicate that American orthodontists typically manage 344 active cases on average every year. By contrast, Taiwanese orthodontists manage fewer cases, presumably because general practitioners provide these services.

In terms of risk perceptions and management, 73.94% of Taiwanese orthodontists primarily acquire risk management knowledge through dental conferences, similar to the trends observed in Germany and Italy.^10^^,^^11^ By contrast, only 9.15% of Taiwanese orthodontists utilize online resources and journals; this proportion is significantly lower than that in countries such as Australia, where these resources are crucial.^12^ Although the COVID-19 pandemic has led to an increasing trend in Taiwanese orthodontists attending online academic seminars, Taiwanese orthodontists still prefer in-person conferences for obtaining information on risk management. Therefore, expanding the use of online platforms for risk management education represents a promising future direction in the field. In terms of continual education, 46.48% of Taiwanese orthodontists report no participation; this proportion is lower than that in several European countries, including the Netherlands, where continual education is mandatory for license renewal.^13^ Taiwanese orthodontists have yet to fully recognize the importance of risk management, underscoring the need for enhanced education in risk management.^7^

In this study, 86.62% of the Taiwanese orthodontists identified patient dissatisfaction with orthodontic outcomes as the most common adverse event, which may have led to monetary settlements. This finding is consistent with those from South Korea, where a discrepancy between patient expectations and treatment outcomes often leads to complaints.^14^ In addition, more than 72% of the Taiwanese orthodontists were aware of the legal requirement to retain patient records for 7 years, a standard similar to those applied in Japan and Germany.^15^ This finding reflects the high professional standards of Taiwanese orthodontists.

In this study, 69.72% of the Taiwanese orthodontists informed their patients of all potential risks, and 95.07% specifically addressed the risks of relapse and retention. These clinical practices align with international practices. For example, in the United States, approximately 70% of orthodontists provide comprehensive information on risks.^16^ Similarly, 60% of orthodontists in Spain consistently disclose all risks.^17^ These findings indicate regional differences in risk communication. In the current study, 70.42% of the Taiwanese orthodontists obtained informed consent from every patient, reflecting strong adherence to legal and ethical standards. This practice is comparable to that in the United Kingdom, in which orthodontists emphasize informed consent.^18^ Moreover, 55% of orthodontists in Brazil consistently acquire informed consent from their patients.^19^ These results indicate variability in obtaining informed consent across countries. In the present study, 80.99% of the Taiwanese orthodontists collected data both before and after treatment, which is crucial for documenting the orthodontic process and monitoring changes, thereby ensuring the retention of valuable medical records. By contrast, approximately 70% of German orthodontists consistently maintain such records.^20^ Data collection is a fundamental practice in risk management, providing a basis to prevent and resolve future disputes. Although the majority of Taiwanese orthodontists collect data, approximately 20% do not, highlighting an area of improvement.

In cases of unsuitable aligner therapy, 92.96% of the Taiwanese orthodontists switched to fixed appliances, indicating their proficiency in managing complications associated with aligners. This rate is higher than those in the United States (85%)^21^ and the Netherlands (78%),^10^ where a more conservative approach is applied. Adjusting treatment plans in response to complications is the optimal approach for preventing potential risks.

In this study, 30.28% of the Taiwanese orthodontists encountered adverse events, and 17% reported monetary settlements. These rates are significantly lower than the adverse event rate of 45% and the settlement rate of 25% reported in the United States.^21^ Compared with their counterparts, American patients have higher awareness and a higher frequency of litigation; thus, American orthodontists encounter higher rates of adverse events. Most of the American orthodontists have malpractice insurance, which is commonly used to manage the financial aspects of adverse event resolution. In the present study, 38.73% of the Taiwanese orthodontists encountered cases of foreign body ingestion, a rate higher than that reported in Spain (25%).^17^

In terms of misdiagnoses or incorrect treatment plans by other orthodontists, 54.93% of the Taiwanese orthodontists informed their patients that their treatment was incomplete, and 88.73% referred their patients back to their original orthodontist. These findings reflect strong professional collaboration among Taiwanese orthodontists for completing patient treatment. These proportions are notably higher than that reported in Germany (70%).^20^ In the present study, 26.06% of the Taiwanese orthodontists did not participate in dental insurance, compared with 35% in Italy.^21^ In conclusion, although adverse events occur, Taiwanese orthodontists demonstrate technical proficiency and collaborative capabilities, leading to a reduced need for monetary settlements or legal resolution. The majority of orthodontists participate in dental insurance, indicating the maturity of orthodontic practices in Taiwan.

Correlation and regression analyses revealed no significant relationship between risk management and the majority of demographic factors in Taiwanese orthodontists. Notably, a significant positive correlation was observed for the frequency of monetary settlements with increasing age (P = 0.028), clinical experience (P = 0.002), and active treatment cases (P = 0.021). This tendency may result from concerns over the potential repercussions of litigation and the associated impact of prolonged litigation.

Multiple regression analysis revealed no significant differences in the majority of demographic factors. Significant correlations were observed for risk management for adverse events with employment status (P = 0.027) and number of active treatment cases (P = 0.010). Similarly, significant correlations were observed for informing patients of potential risks with clinical experience (P = 0.045), employment status (P = 0.020), and number of active treatment cases (P = 0.002). These results indicated that orthodontists who had more active cases and were self-employed encountered more adverse events and exhibited a stronger tendency to inform their patients of potential risks. Dental clinic employers, who bear full responsibility for any errors occurring in their clinics, may place a greater emphasis on risk management compared with employed orthodontists. Similarly, orthodontists with more active treatment cases are more concerned about potential errors; therefore, they tend to prioritize risk management practices more attentively. Overall, these findings indicate that Taiwanese orthodontists adhere to risk management protocols, regardless of their demographics.

Orthodontists in Taiwan have a positive understanding of and attitude toward risk management, and they exhibit minimal differences in their perceptions, attitudes, and experiences of risk management. These results suggest that the majority of Taiwanese orthodontists adhere to risk management practices, regardless of their demographics. However, the percentages of orthodontists who participate in dental insurance and receive continual education on risk management are low. Therefore, governmental organizations should implement policies aimed at ensuring that Taiwanese orthodontists receive continual education on risk managements.

## Declaration of competing interest

The authors have no conflicts of interest relevant to this article.
